# Chemotherapy in advanced biliary tract carcinoma: a pooled analysis of clinical trials

**DOI:** 10.1038/sj.bjc.6603648

**Published:** 2007-02-27

**Authors:** F Eckel, R M Schmid

**Affiliations:** 1 Department of Internal Medicine II, Klinikum rechts der Isar, Technical University of Munich, Munich, Germany

**Keywords:** bile duct carcinoma, biliary tract carcinoma, cholangiocarcinoma, gallbladder carcinoma, gemcitabine, platinum compounds

## Abstract

Owing to the lack of randomised controlled trials no standard of chemotherapy exists in the treatment of advanced biliary tract carcinoma. 5-fluorouracil or gemcitabine is recommended based on small and predominately phase II trials. The aim of this analysis was to analyse existing trials, even small and nonrandomised, and identify superior regimens. Chemotherapy trials published in English from 1985 to July 2006 were analysed as well as ASCO abstracts from 1999 to 2006. Response rate (RR=CR+PR), tumour control rate (TCR=CR+PR+SD), time to tumour progression (TTP), overall survival (OS), and toxicity were analysed. One hundred and four trials comprising 112 trial arms and 2810 patients, thereof 634 responders and 1368 patients with tumour control were analysed. Pooled RR and TCR were 22.6 and 57.3%, respectively. Significant correlations of RR and TCR with survival times were found. Subgroup analysis showed superior RRs for gallbladder carcinoma (GBC) compared with cholangiocarcinoma, but shorter OS for GBC. Furthermore, superior RRs and TCRs of gemcitabine and platinum containing regimens were found with highest RRs and TCRs in the combination subgroup. Based on published results of predominately phase II trials, gemcitabine combined with platinum compounds represents the provisional standard of chemotherapy in advanced biliary tract cancer, unless a new evidence-based standard has been defined.

Biliary tract carcinomas (BTC) are uncommon but highly fatal malignancies in the United States and Europe. BTC comprise gallbladder carcinoma (GBC) and cholangiocarcinoma (CC) (bile duct cancer), which arise from the epithelial cells of the intrahepatic and extrahepatic bile ducts. The anatomic location of CC can be described as intrahepatic, distal extrahepatic, or hilar. Lesions can be described as mass-forming, periductal or intraductal, or as mixed mass-forming and periductal (Patel, 2006).

Approximately 5000 cases of GBC and 2500 cases of CC are diagnosed annually in the USA (de Groen *et al*, 1999). The incidence of CC (particularly, intrahepatic CC) has been rising over the past two decades in the United States, United Kingdom, and Australia (Rajagopalan *et al*, 2004). Worldwide, the highest prevalence of GBC is seen in India, Pakistan, Ecuador, Israel, Mexico, Chile, Japan, and among Native American women, particularly those living in New Mexico. Mortality rates in these areas can reach 5–10 times that in the United States (Lazcano-Ponce *et al*, 2001; Randi *et al*, 2006). Worldwide, CC accounts for 3% of all gastrointestinal cancers and is the second commonest primary hepatic tumour (Khan *et al*, 2005). Incidence of CC is highest in Israel, Japan, among Native Americans, and in Southeast Asia, where it can reach 87 per 100 000 (Rajagopalan *et al*, 2004). This indicates the global significance of both GBC and CC.

The reported incidence of ‘surprise’ or ‘incidental’ GBC varies from 0.35 to 2% (Misra and Guleria, 2006). Even in patients undergoing aggressive surgery, the general outcome of patients with BTC has been disappointing. Five-year survival rates are 5-10% for GBC and 10–40% for CC (de Groen *et al*, 1999). Unfortunately, most biliary tract carcinomas are diagnosed at advanced stages when the tumour is unresectable. Median survival of patients with advanced disease is in the range of only a few months.

Owing to the lack of randomised phase III studies, there is no standard regimen for palliative chemotherapy of GBC and CC. Depending on the patient's general condition best supportive care, a clinical trial, 5-fluorouracil, or gemcitabine is recommended according to guidelines of the National Comprehensive Cancer Network.

The aim of this study was to extensively analyse existing data of published clinical trials, even small and non-randomised, and, if possible, identify superior regimens, which may represent a standard of care of palliative chemotherapy in this disease.

## METHODS

Data for this analysis were identified by searches of PubMed and references from relevant articles using the search terms ‘biliary tract neoplasms’, ‘bile duct neoplasms’, ‘cholangiocarcinoma’, and ‘gallbladder neoplasms’. Only papers reporting the results of chemotherapy trials published in English from January 1985 to July 2006 were included. Abstracts from ASCO meetings presented from 1999 to 2006 were included also. Trials of intra-arterial hepatic chemotherapy or of chemoradiotherapy were excluded.

For inclusion of a trial in the analysis, the number of patients (included, treated, or evaluable) and the response rate (RR=CR+PR) were required at least. Furthermore, tumour control rate (TCR=CR+PR+SD), time to tumour progression (TTP), and overall survival (OS) were recorded if available. In trials with more than one treatment arm, arms were analysed separately as single-arm trials. All trials included were analysed independent of the tumour classification for BTC. In addition, subgroup analysis was performed for GBC-only and CC-only for trials with sufficient data.

For subgroup analysis results of the trials were compared and tested nonparametrically (Mann–Whitney, Kruskal–Wallis). Furthermore, RR and TCR data were pooled by summarising the number of patients of the trials. For example, a pooled RR was computed as the sum of the responders of a subgroup divided by the sum of the patients of this subgroup. Ninety-five per cent confidence interval (CI) was calculated by the method of Clopper and Pearson. Proportions (e.g., pooled RRs) were compared by *z*-test. Nonparametric Correlation was tested according to Spearman.

## RESULTS

One hundred and four trials comprising of 112 trial arms were included in this analysis (for references see Appendix A). Only three were randomised trials, thereof two phase II (Kornek *et al*, 2004; Ducreux *et al*, 2005) and one phase III (Rao *et al*, 2005). No appropriate trial could be identified as published 1992 or earlier (limited to 1985). Seventeen (15%) trials were published from 1993 to 1999, whereas 95 (85%) trials were published from 2000 to July 2006. The 112 trials analysed comprise a total of 2810 patients treated.

The number of patients per trial ranged from 5 to 65 resulting in a mean number of patients per trial of 25.1 with a small range in various subgroups. The mean number of patients per trial (or per subgroup of a trial) for GBC-only and CC-only was smaller with 16.7 and 19.6, respectively.

**Figure 1:**
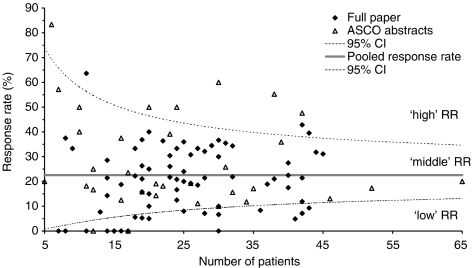
Response rates of all trials analysed sorted by the number of patients. Full papers are indicated by black rhombi and ASCO abstracts by empty triangles. The horizontal grey line represents the pooled response rate of all patients (22.6%). The limits of the 95% CI of the overall pooled RR are shown by doted lines.

**Figure 2:**
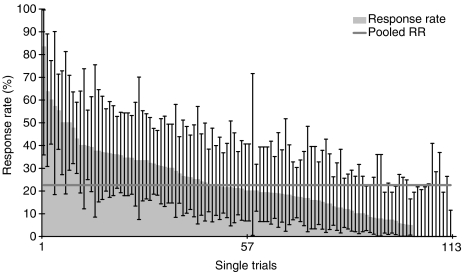
Response rate and 95% CI of all trials analysed sorted by the RR. The horizontal grey represents the pooled RR of all patients (22.6%).

Among all 2810 patients (25.1 per trial) analysed, 634 responders (5.7 per trial) were observed resulting in a pooled RR of 22.6% (95% CI 21.0–24.2%, *n*=2810). The RRs of all trials analysed sorted by the number of patients are shown in Figure 1. The RR of nine (8%) trials was above the upper limit of the 95% CI of 22.6% (Figure 1, ‘high’ RR). Twenty two (20%) trials had RRs below the lower limit of the 95% CI of 22.6% (‘low’ RR) and 81 (72%) trials had RRs in the range of the 95% CI (‘middle’ RR). The nine ‘high’ RR trials evaluated gemcitabine plus platinum compounds (*n*=5), fluoropyrimidines plus platinum compounds (*n*=3), and gemcitabine alone (*n*=1). Among the 22 trials with ‘low’ RRs are trials evaluating docetaxel, paclitaxel, irinotecan, gemcitabine, and fluoropyrimidines as well as new drugs (erlotinib, lapatinib, exatecan, dolastatin, lanreotide). Figure 2 shows the RR and its 95% CI of all trials analysed sorted by the RR.

Ninety six trials reported stable disease or TCR data. These 96 trials comprise 2386 patients, thereof 1368 patients achieving tumour control (14.3 patients with tumour control per trial) resulting in a pooled TCR of 57.3% (95% CI 55.3–59.3%, *n*=2386).

### Survival

**Figure 3:**
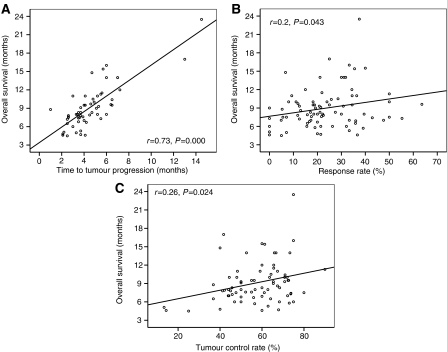
(**A**–**C**) Charts showing the correlation between RR, TCR, TTP and OS.

The median TTP and OS for all patients was 4.1 months (60 trials, 1543 patients) and 8.2 months (82 trials, 2197 patients), respectively. There was a highly significant correlation between RR and TCR (*r*=0.59, *P*=0.000), RR and TTP (*r*=0.52, *P*=0.000), TCR and TTP (*r*=0.66, *P*=0.000), and TTP and OS (Figure 3A). Furthermore, a significant weak correlation between RR and OS as well as TCR and OS was found (Figures 3B and C). Regression equation showed a 10% increment in RR corresponding to an 8% increase of TCR, a 0.7-month increase of TTP, and a 0.6-month increase of OS. A 10% increment in TCR corresponded to a 0.7-month increase of TTP, a 0.7-month increase of OS, whereas a 1-month increase in TTP corresponded to a 1.3-month increase of OS.

### Subgroups

The RR of trials (subgroups) of patients with GBC was higher compared with CC (number of patients 500 *vs* 471, pooled RR 34.4 *vs* 20.2%, *P*=0.000; median RR of trials 35.5 *vs* 17.7%, *P*=0.008). For the TCR, there was no significant difference between GBC and CC (pooled TCR 60.5 *vs* 59.7%, *P*=0.904; median RR of trials 60.0 *vs* 55.0%, *P*=0.784). In contrast, the OS was significantly longer in trials (subgroups) of patients with CC compared with GBC (median 9.3 *vs* 7.2 months, *P*=0.048).

Comparison of regimens containing one or two drugs showed significant superiority of two drug combinations compared with monotherapy concerning RR (number of patients 1499 *vs* 971, pooled RR 28.0 *vs* 15.3%, *P*=0.000; median RR of trials 25.8 *vs* 11.8%, *P*=0.000), TCR (pooled TCR 61.0 *vs* 50.4%, *P*=0.000; median TCR of trials 60.0 *vs* 48.0%, *P*=0.003), and TTP (median 4.4 *vs* 3.4 months, *P*=0.015) with a trend for OS (median 9.3 *vs* 7.5 months, *P*=0.061). Polychemotherapy (three or more drug regimens) resulted in a lower RR compared with two drug combinations (number of patients 340 *vs* 1499, pooled RR 19.1 *vs* 28.0%, *P*=0.000; median RR of trials 19.2 *vs* 25.8%, *P*=0.065) but no difference in OS (median 9.0 *vs* 9.3 months). Comparison of polychemotherapy with monotherapy showed higher TCR (pooled TCR 58.9 *vs* 50.4%, *P*=0.028; median TCR of trials 62.8 *vs* 48.0%, *P*=0.098), longer TTP (median 5.2 *vs* 3.4 months, *P*=0.016), and OS (median 9.0 *vs* 7.5 months, *P*=0.086) of multiple drug combinations.

**Figure 4:**
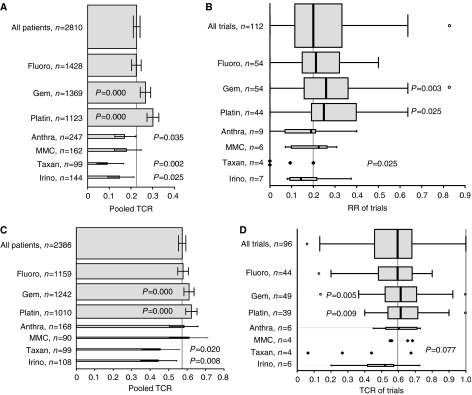
(**A**–**D**) Fluoro: fluoropyrimidines (fluorouracil, capecitabine, tegafur); Gem: gemcitabine; Platin: platinum compounds (cisplatin, oxaliplatin, carboplatin); Anthra: anthracyclines (adriamycin, epirubicin); MMC: mitomycin C; Taxan: taxanes (paclitaxel, docetaxel), Irino: irinotecan. (**A**) Pooled RRs (RR=CR+PR) and 95% CIs of all patients included in the analysis and of subgroups of patients, defined by treatment with regimens containing a particular drug regardless of other drugs. The height of the bars correlates with the number of patients. The *P*-values apply to the comparison of a subgroup, defined by a particular drug *vs* all other patients, which were not treated with this drug (e.g., patients treated with gemcitabine or gemcitabine-containing combinations *vs* patients treated with gemcitabine-free regimens). The RRs of the comparison subgroups are not shown. The vertical grey line represents the pooled RR of all patients (pts, 22.6%). (**B**) Boxplots of the RRs of all trials and of subgroups, defined by a particular drug. The height of the boxplots correlates with the number of trials. *P*-values for subgroup comparison as in Figure 3A. The vertical grey line represents the median RR of all trials (20.0%). For subgroups consisting of less than five trials, results of single trial are shown and no boxplots are provided. (**C**) Pooled TCRs (TCR=CR+PR+SD) and 95% CIs as in Figure 3A. The vertical grey line represents the pooled TCR of all patients (57.3%). (**D**) Boxplots of the TCRs as in Figure 3B. The vertical grey line represents the median TCR of all trials (59.6%).

Further subgroup analysis focused on cytotoxic agents. Subgroups of patients treated with regimens containing a particular drug were compared with all other patients, who were treated with regimens, that did not contain this particular drug, regardless of other drugs. Subgroups were defined by fluoropyrimidines (fluorouracil, capecitabine, tegafur), gemcitabine, platinum compounds (cisplatin, oxaliplatin, carboplatin), anthracyclines (adriamycin, epirubicin), mitomycin C, taxanes (paclitaxel, docetaxel), and irinotecan. RR and TCR were analysed by pooling all patients (bars with 95% CI) as well as all trials (boxplots) (Figure 4A–D). Results of treatment with fluoropyrimidines were very similar to the results of all fluoropyrimidine-free regimens and may represent the results of all patients and all trials analysed. In contrast, treatment with gemcitabine as well as with platinum compounds resulted consistently in highly significant superior RRs and TCRs compared with gemcitabine-free as well as to platinum-free combinations (Figure 4A–D). In contrast, beside from a trend for a longer TTP of the gemcitabine subgroup (4.6 *vs* 3.7 months, *P*=0.085), differences in survival times were small (platin *vs* no platin: TTP and OS 0.7 months each) and not significant.

**Figure 5:**
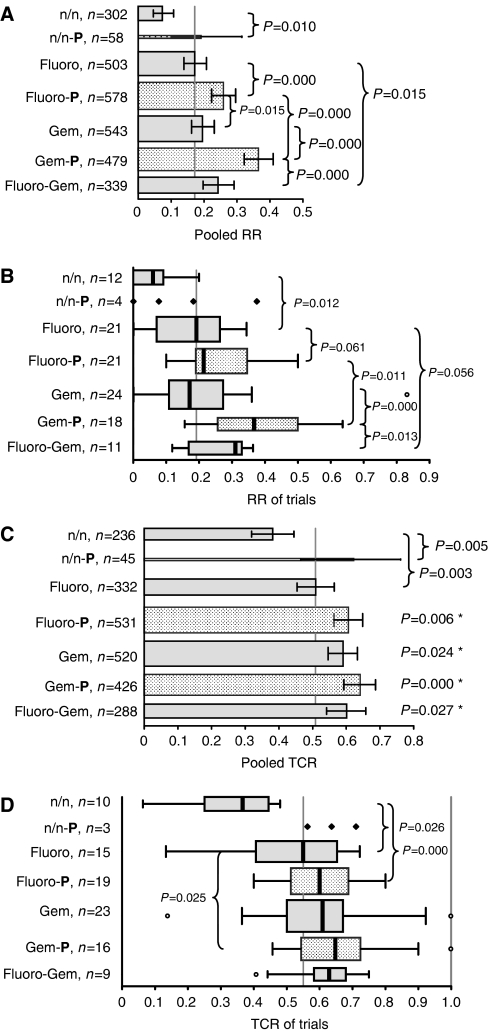
(**A**–**D**) Fluoro: n/n: neither Fluoro nor Gem; Fluoro: fluoropyrimidines (fluorouracil, capecitabine, tegafur); Gem: gemcitabine; P: platinum compounds (cisplatin, oxaliplatin, carboplatin). (**A**) Pooled RRs (RR=CR+PR) and 95% CIs of subgroups of patients, defined by treatment with fluoropyrimidines, gemcitabine, and platinum compounds, regardless of other drugs. The height of the bars correlates with the number of patients. The vertical grey line represents the pooled RR of the Fluoro subgroup (17.1%). The Fluoro-Gem-P subgroup consists of only eight patients and is therefore not shown. Additional *P*-values: Fluoro *vs* Gem-P: 0.000; n/n *vs* all other subgroups: 0.000; n/n-P *vs* Gem-P: 0.012. (**B**) Boxplots of RRs of subgroups of trials, defined by treatment with fluoropyrimidines, gemcitabine, and platinum compounds. The height of he boxplots correlates with the number of trials. The vertical grey line represents the median RR of the Fluoro subgroup (19.2%). The Fluoro-Gem-P subgroup consists of only one trial and is therefore not shown. For subgroups consisting of less than five trials, results of single trial are shown and no boxplots are provided. Additional *P*-values: Fluoro *vs* Gem-P: 0.000; Fluoro-P *vs* Gem: 0.033; n/n *vs* all other subgroups: ⩽0.002; n/n-P *vs* Gem-P: 0.042. (**C**) Pooled TCRs (TCR=CR+PR+SD) and 95% CIs of subgroups of patients as in Figure 4A. The vertical grey line represents the pooled TCR of the Fluoro subgroup (50.9%). ^*^P-value in comparison to the Fluoro subgroup. Additional *P*-values: n/n *vs* all other subgroups: 0.000. (**D**) Boxplots of TCRs of subgroups of trials as in Figure 4C. The vertical grey line represents the median RR of the Fluoro subgroup (55.0%). Additional *P*-values: n/n *vs* all other subgroups: ⩽0.003.

For further investigation of the effects of fluoropyrimidines, gemcitabine, and platinum compounds, subgroups defined by treatment with these three agents and all combinations (regardless of other drugs) were analysed considering RR and TCR for all patients and all trials (Figure 5A–D). As shown in Figure 5A–B the RR of treatment with gemcitabine was not significantly higher compared with fluoropyrimidines. The addition of platinum compounds increased the RR of fluoropyrimidines as well as of gemcitabine. The increase of the RR by the addition of platinum compounds to gemcitabine was double the increase of the addition to fluoropyrimidines (17.0 *vs* 8.7%). The increase of the RR by the addition of gemcitabine to fluoropyrimidines was similar to the addition of platinum compounds to fluoropyrimidines.

In contrast to analysation for RR, pooled TCR of the gemcitabine subgroup was significantly higher compared with fluoropyrimidines (*P*=0.024, Figure 5C). The addition of platinum compounds to fluoropyrimidines and gemcitabine increased the TCRs, but the difference was significant for the pooled TCR of the fluoropyrimidine subgroup only (9.7%, *P*=0.006). Just as for RRs, TCR was highest in the gemcitabine–platinum combination subgroup. Compared with the fluoropyrimidines subgroup the difference was significant for the pooled TCR (*P*=0.000, Figure 5C) as well as for the median TCR (*P*=0.025, Figure 5D). There was a trend for a longer TTP in the gemcitabine–platinum combination subgroup compared with the fluoropyrimidines–platinum combination subgroup (5.5 *vs* 3.7 months, *P*=0.072, 21 trials). All other differences of TTP and OS between subgroups were small and not statistically significant.

There were only a few trials evaluating new drugs, such as erlotinib, lapatinib, dolastatin, exatecan, rebeccamycin (one trial each, monotherapy), and raltitrexed (two trials, combination with gemcitabine and cisplatin/epirubicin, respectively). For separate analysis of a new agent subgroup, separately the numbers of trials and patients are too low. Monotherapy trials of new agents are subsumed in the *n*/*n* subgroup (neither fluoropyrimidine nor gemcitabine without platinum compounds, Figure 5A–D).

### Statistics

Only a minority of the trials reported statistical considerations such as sample size calculation, null and alternative hypothesis, significance level, and power. The preferred test design was the Simon two-stage design. Significance level (alpha) was mostly 0.05 (range 0.03–0.10) and the power was mostly 80% (range 80% – 95%). The null hypotheses tested ranged from an RR of *p*_0_ ⩽5 to ⩽20% with alternative hypotheses between an RR of *p*_A_⩾15 and ⩾40%. The number of trials analysed in this study, which would have been negative, if tested with RR or TCR as primary end point against different alternative hypotheses p_A_ with different powers, are listed in Table 1A, whereas Table 1B shows the number of trials, which would have been positive if tested against different null hypotheses *p*_0_ with different significance levels (alpha).

## DISCUSSION

This pooled analysis of all published clinical trials since 1985 showed that chemotherapy with gemcitabine combined with cisplatin or oxaliplatin increases RR and TCR in GBC and CC. Our findings provide best possible evidence that this combination chemotherapy may improve survival in these diseases.

This is the first systematic review including a comprehensive statistical analysis of advanced GBC and CC. One hundred and four trials comprising 112 trial arms were included in this analysis. Pooled RR of all patients was 22.6% (95% CI 21.0–24.2%). RRs of single trials range from 0% to more than 80% and the median RR was 20.0% with a first and third quartile of 11.5 and 33.2%, respectively. In other words, one-fourth of all trials reported RRs less than or equal to 11.5% and another fourth RRs greater than or equal to 33.2%. The aims of this analysis were to identifiy superior regimens among this extreme range of RRs and thus to provide a standard of chemotherapy in advanced BTC, even based on phase II trials before the background of missing phase III trials.

The cochrane collaboration published a protocol to assess the beneficial and harmful effects of chemotherapy for gallbladder cancer (Pandey and Krishnan, 2004). Initially, the review was expected to be published in Issue 4, 2005. However, owing to the principles of the cochrane collaboration, this review should address randomised trials evaluating chemotherapy *vs* placebo/no chemotherapy and one type of chemotherapy *vs* another type of chemotherapy. As almost no randomised trials exist, this cochrane review will not be finished at all.

Guidelines for the treatment of CC have been published 2002 by the BASL (British Association for the Study of the Liver) (Khan *et al*, 2002). Consensus conclusion from predominately phase II trials suggest: (i) RRs is of 5-fluorouracil based and (older) single agents is 10–20%, (ii) RRs of newer single agents, such as gemcitabine, vary from 20 to 30%, (iii) RRs of recent phase II combinations vary from 20 to 40%, and (iv) gemcitabine in combination with cisplatin shows 30–50% RRs. The results of the present analysis are somewhat different, but agree in principle concerning the combination of gemcitabine with cisplatin: (i) more than a half of fluoropyrimidine-based trials reported RRs of more than 20%, (ii) more than a half of single-agent gemcitabine trials and nearly all trials of newer single agents reported RRs of less than 20%, (iii) about 40% of combination trials reported RRs of 20% or less, and (iv) the middle half of gemcitabine plus platinum combinations results in RRs between 26 and 50%, that is, one-quarter of this combination trials reported RRs of 50% or greater (Figure 5B).

Three randomised trials, thereof only one phase III, were included in the present study. The 40955 EORTC phase II trial compared high-dose 5-FU with a combination of cisplatin, 5-FU, and folinic acid (Ducreux *et al*, 2005). The RR was higher in the combination arm (19 *vs* 7%), but there was no difference concerning disease stabilisation and toxicity was increased. Based on potential drug synergy a phase II trial compared two experimental arms: MMC combined with biweekly high-dose gemcitabine *vs* MMC combined with capecitabine (Kornek *et al*, 2004). The latter combination resulted in higher RR (31 *vs* 20%), TTP (5.3 *vs* 4.2 months), and OS (9.3 *vs* 6.7 months). A statistical comparison of the two groups including *P*-values was not published. The authors conclude that MMC combined with capecitabine seems to be superior, and further evaluation seems warranted. The only phase III trial of the present analysis compared etoposide, 5-FU, and folinic acid with epirubicin, cisplatin, and 5-FU (ECF) (Rao *et al*, 2005). As a result of poor recruitment (*n*=54) the trial was underpowered to detect a significant difference in OS. The ECF regimen was associated with less toxicity, but in conclusion, based on these data it is not possible to define a reference regimen for advanced BTC.

Owing to the lack of randomised phase III trials, there is a need to define treatment standards on predominately phase II trials. For this reason, we will necessarily act on imperfect evidence. This issue was discussed recently (Djulbegovic *et al*, 2005). For a treatment goal of prolongation of survival by days to months, highest standards of experimental evidence (well-designed and large-scale conducted RCTs) were proposed. The increasing number of publications of chemotherapy trials in this disease emphasise the need of a new standard beyond 5-FU. Hopefully, the increasing number of publications will be followed by an increasing quality of the trials. Only a minority of the trials analysed published statistical considerations and frequently results were subsumed as being promising. For well designed phase II trials it is necessary to prospectively define a null hypothesis, an alternative hypothesis, the significance level (alpha), and the power (Tables 1A, B). By the use of the Simon MinMax two-stage design and reasonable parameters, the number of patients of a phase II trial will not exceed a total of 40 patients and 25 patients for the first stage.

The longer TTP and OS of multiple drug combinations may be due to more strict inclusion criteria of potentially more toxic regimens and may indicate selection bias. Furthermore, the proportion of patients with different localisations of their cancers may contribute to selection bias, as the present analysis showed higher RRs but shorter OS of GBC compared with CC.

Subgroup analysis concerning the three most important drugs demonstrated that gemcitabine alone is not superior to fluoropyrimidines. Platinum compounds increase the activity of both fluoropyrimidines and gemcitabine. The increase of the addition of platinum compounds to gemcitabine is greater compared with the addition to fluoropyrimidines. Synergism of cisplatin and gemcitabine has been demonstrated in cell lines and is based on direct inhibitory effect of gemcitabine on the repair of cisplatin interstrand adducts and interstrand crosslinks (van Moorsel *et al*, 1999; Moufarij *et al*, 2003).

The present analysis demonstrated gemcitabine combined with platinum compounds superior concerning both RR and TCR. As RR and TCR significantly correlate with survival times (TTP and OS), RR and TCR represent a meaningful surrogate in BTC. In patients with colorectal cancer a meta-analysis of randomised phase III trials demonstrated highly significant correlation between RR and TTP, TTP and OS, and RR and OS (Louvet *et al*, 2001). A 10% RR increment corresponded to a 1-month increase in TTP and a 0.9-month increase in OS, whereas a 1-month increase in TTP corresponded to a 0.7-month increase of OS in colorectal cancer patients on first-line treatment. Our findings in BTC demonstrated a 10% RR increase corresponding to a 0.7-month increase in TTP and a 0.6-month increase in OS, whereas a 1-month increase in TTP corresponded to a 1.3-month increase of OS. Consequently, the data of highest experimental evidence in colorectal cancer confirm the results of our pooled analysis of clinical and predominately phase II trials in BTC.

The evidence level of this pooled analysis is limited as discussed above and it remains unclear which platinum compound is optimal and what schedule of administration should be used. Therefore, it is essential to perform randomised trials, such as the UK National Cancer Research Institute ABC-02 trial, to evaluate the definite role of platinum compounds in combination with gemcitabine compared with gemcitabine alone. This and similar trials are needed to establish reference regimens for this disease.

In conclusion, we suggest gemcitabine combined with cisplatin or oxaliplatin as the most active, and therefore a provisional standard regimen in BTC until a new evidence-based standard is defined.

## Figures and Tables

**Table 1:** Number of (A) negative trials and (B) positive trials

**(A) Number of negative trials^a^**					
**RR (*n*=112)**	**Power**		**TCR (*n*=96)**	**Power**	
**H_A_: *p*_A_**	**80%**	**90%**	**H_A_: *p*_A_**	**80%**	**90%**
⩾22.6%	32 (29%)	25(22%)	⩾57.3%	25 (26%)	15 (16%)
⩾30.0%	56 (50%)	40(36%)	⩾65.0%	36 (38%)	31 (32%)
⩾35.0%	66 (59%)	58(52%)	⩾70.0%	52 (54%)	39 (41%)
⩾40.0%	77 (69%)	67(60%)	⩾75.0%	69 (72%)	53 (55%)
					
**(B) Number of positive trials^b^**					
**RR (*n*=112)**	**Alpha**		**TCR (*n*=96)**	**Alpha**	
**H_0_: *p*_0_**	**0.05**	**0.10**	**H_0_: *p*_0_**	**0.05**	**0.10**
⩽10.0%	47 (42%)	58 (52%)	⩽45.0%	35 (36%)	43 (45%)
⩽15.0%	35 (31%)	36 (32%)	⩽50.0%	21 (22%)	33 (34%)
⩽20.0%	21 (19%)	28 (25%)	⩽55.0%	11 (11%)	18 (19%)
⩽22.6%	10 (9%)	20 (18%)	⩽57.3%	9 (9%)	13 (14%)

RR=Response Rate; TCR=Tumour Control Rate.

a Number of trials analysed, which would have been negative, if tested with (RR or TCR as primary end point against four different alternative hypotheses *p*_A_ with two different powers.

b Number of trials analysed, which would have been positive, if tested with (RR) or TCR as primary end point against four different null hypotheses *p*_0_ with two different significance levels (alpha).

## Appendix A

References of the trials included in this analysis: Abid L, *et al* (2003). *Proc ASCO* **22**: abstr. 1302. Abou-Alfa GK, *et al* (2005). *Am J Clin Oncol* **28**: 334–339. Alberts SR, *et al* (2005). *Cancer* **103**: 111–1118. Alberts SR, *et al* (2002). *Int J Gastrointest Cancer* **32**: 107–114. Andre T, *et al* (2004). *Ann Oncol* **15**: 1339–1343. Arroyo G, *et al* (2001). *Proc ASCO* **20**, abstr 626. Baluch S, *et al* (2003). *Proc ASCO* **22**: abstr 1473. Bhargava P, *et al* (2003). *Oncology (Huntingt)* **17**: 23–26. Carraro S, *et al* (2003). *Proc ASCO* **20**: abstr 2333. Chang H, *et al* (2005). *Proc ASCO* **23**: abstr 4173. Chen JS, *et al* (1998). *Anticancer Drugs* **9**, 393–397. Chen JS, *et al* (2001). *Anticancer Drugs* **12**: 339–343. Chen JS, *et al* (2003). *Jpn J Clin Oncol* **33**: 353–356. Cho JY, *et al* (2005a). *Yonsei Med J* **46**: 526–531. Cho JY, *et al* (2005b). *Cancer* **104**: 2753–2758. Choi CW, *et al* (2000). *Am J Clin Oncol* **23**: 425–428. Dobrilla-Dintinjana R, *et al* (2005). *Proc ASCO* **23**: abstr 4268. Doval DC, *et al* (2004). *Br J Cancer* **90**: 1516–1520. Dowlati A, *et al* (2003). *Proc ASCO* **22**: abstr 1070. Duck L, *et al* (2002). *Proc ASCO* **21**: abstr 2314. Ducreux M, *et al* (1998). *Ann Oncol* **9**, 653–656. Ducreux M, *et al* (2005). *Eur J Cancer* **41**: 398–403. Eckel F, *et al* (2000). *Ann Oncol* **11**: 762–763. Ellis PA, *et al* (1995). *Eur J Cancer* **31A**, 1594–1598. Eng C, *et al* (2004). *Am J Clin Oncol* **27**: 565–569. Feisthammel J, *et al* (2006). *Proc ASCO* **24**: abstr 14088. Ferrari VD, *et al* (2004). *Am J Clin Oncol* **27**: 445–448. Fiebiger WC, *et al* (2002). *Scand J Gastroenterol* **37**: 222–225. Gallardo JO, *et al* (2001). *Ann Oncol* **12**: 1403–1406. Gebbia N, *et al* (2005). *Proc ASCO* **23**: abstr 4132. Gebbia V, *et al* (2001). *J Clin Oncol* **19**: 4089–4091. Gebbia V, *et al* (1996). *Cancer* **78**: 1300–1307. Gelibter A, *et al* (2005). *Cancer* **104**: 1237–1245. Glover KY, *et al* (2005). *Proc ASCO* **23**: abstr 4123. Harder J, *et al* (2006). *Proc ASCO* **24**: abstr 14006. Hegewisch-Becker S, *et al* (2003). *Proc ASCO* **22**: abstr 1247. Hsu C, *et al* (2004). *Br J Cancer* **90**: 1715–1719. Ikeda M, *et al* (2005). *Jpn J Clin Oncol* **35**: 439–443. Iqbal, S, *et al* (2006). *Proc ASCO* **24**: abstr 4134. Iyer RV, *et al* (2005). *Proc ASCO* **23**: abstr 4230. Jones DV, Jr, *et al* (1996). *J Clin Oncol* **14**: 2306–2310. Julka PK, *et al* (2006). *Hepatobiliary Pancreat Dis Int* **5**: 110–114. Kajanti M & Pyrhonen S (1994). *Am J Clin Oncol* **17**: 223–226. Kiba T, *et al* (2006). *Proc ASCO* **24**: abstr 14122. Kim ST, *et al* (2006). *Cancer* **106**: 1339–1346. Kim TW, *et al* (2002). *Proc ASCO* **21**: abstr 2273. Kim TW, *et al* (2003). *Ann Oncol* **14**: 1115–1120. Kindler HL, *et al* (2005). *Invest New Drugs* **23**: 489–493. Knox JJ, *et al* (2005). *J Clin Oncol* **23**: 2332–2338. Knox JJ, *et al* (2004). *Ann Oncol* **15**: 770–774. Kobayashi K, *et al* (2006). *BMC Cancer* **6**, 121. Kornek GV, *et al* (2004). *Ann Oncol* **15**: 478–483. Kubicka S, *et al* (2001). *Hepatogastroenterology* **48**: 783–789. Kuhn R, *et al* (2002). *Invest New Drugs* **20**: 351–356. Lee F, *et al* (2006a). *Proc ASCO* **24**: abstr 14126. Lee GW, *et al* (2006b). *Am J Clin Oncol* **29**: 127–131. Lee MA, *et al* (2004). *J Cancer Res Clin Oncol* **130**: 346–350. Lim JY, *et al* (2006). *Proc ASCO* **24**: abstr 14093. Lin MH, *et al* (2003). *Chemotherapy* **49**: 154–158. Lozano R, *et al* (2000). *Proc ASCO* **19**: abstr 1025. Malik IA & Aziz Z (2003). *Am J Clin Oncol* **26**: 124–126. Malik IA, *et al* (2003). *Am J Clin Oncol* **26**: 174–177. Mehrotra B, *et al* (2004). *Proc ASCO* **22**: abstr 4259. Mezger J, *et al* (1998). *Onkologie* **21**: 232–234. Misra S, *et al* (2005). *Proc ASCO* **23**: abstr 4136. Morizane C, *et al* (2003). *Oncology* **64**: 475–476. Murad AM, *et al* (2003). *Am J Clin Oncol* **26**: 151–154. Nehls O, *et al* (2002). *Br J Cancer* **87**: 702–704. Nehls O, *et al* (2006). *Proc ASCO* **24**: abstr 4136. Okada S, *et al* (1994). *Oncology* **51**: 515–517. Okusaka T, *et al* (2006). *Cancer Chemother Pharmacol* **57**: 647–653. Papakostas P, *et al* (2001). *Eur J Cancer* **37**: 1833–1838. Park JO, *et al* (2005a). *Proc ASCO* **23**: abstr 4133. Park JS, *et al* (2005b). *Jpn J Clin Oncol* **35**: 68–73. Park KH, *et al* (2005c). *Cancer* **103**: 2338–2343. Park SH, *et al* (2006). *Cancer* **106**: 361–365. Patt YZ, *et al* (2004). *Cancer* **101**: 578–586. Patt YZ, *et al* (2001). *Clin Cancer Res* **7**, 3375–3380. Patt YZ, *et al* (1996). *J Clin Oncol* **14**: 2311–2315. Pazdur R, *et al* (1999). *Am J Clin Oncol* **22**: 78–81. Peeters M, *et al* (2003). *Proc ASCO* **22**: abstr 1432. Penz M, *et al* (2001). *Ann Oncol* **12**: 183–186. Philip PA, *et al* (2006). *J Clin Oncol* **24**: 3069–3074. Raderer M, *et al* (1999). *Oncology* **56**: 177–180. Ramanathan RK, *et al* (2006). *Proc ASCO* **24**: abstr 4010. Rao S, *et al* (2005). *Br J Cancer* **92**: 1650–1654. Reyes-Vidal J, *et al* (2003). *Proc ASCO* **22**: abstr 1095. Romano R, *et al* (2004). *Proc ASCO* **22**: abstr 4145. Sanz-Altamira PM, *et al* (1998). *Cancer* **82**: 2321–2325. Sanz-Altamira PM, *et al* (2001). *Ann Oncol* **12**: 501–504. Sharma A, *et al* (2006). *Proc ASCO* **24**: abstr 14027. Sharma A, *et al* (2002). *Proc ASCO* **21**: abstr 2381. Stieler JM, *et al* (2003). *Proc ASCO* **22**: abstr 1233. Taal BG, *et al* (1993). *Ann Oncol* **4**, 607–609. Taieb J, *et al* (2002). *Ann Oncol* **13**: 1192–1196. Takada T, *et al* (1998). *Hepatogastroenterology* **45**: 2020–2026. Thongprasert S, *et al* (2005). *Ann Oncol* **16**: 279–281. Tsavaris, N, *et al* (2004). *Invest New Drugs* **22**: 193–198. Tsuji, A, *et al* (2003). *Proc ASCO* **22**: abstr 1484. Uchida, K, *et al* (2005). *Proc ASCO* **23**: abstr 4239. Ueno, H, *et al* (2004). *Br J Cancer* **91**: 1769–1774. Valencak, J, *et al* (1999). *Onkologie* **22**: 498–501. von Delius, S, *et al* (2005). *BMC Cancer* **5**, 61. Yamashita, Y, *et al* (2006). *Anticancer Res* **26**: 771–775.

## References

[bib1] de Groen PC, Gores GJ, LaRusso NF, Gunderson LL, Nagorney DM (1999) Biliary tract cancers. N Engl J Med 341: 1368–1378 10536130 10.1056/NEJM199910283411807

[bib2] Djulbegovic B, Frohlich A, Bennett CL (2005) Acting on imperfect evidence: how much regret are we ready to accept? J Clin Oncol 23: 6822–6825 16145058 10.1200/JCO.2005.06.007

[bib3] Ducreux M, Van Cutsem E, Van Laethem JL, Gress TM, Jeziorski K, Rougier P, Wagener T, Anak O, Baron B, Nordlinger B (2005) A randomised phase II trial of weekly high-dose 5-fluorouracil with and without folinic acid and cisplatin in patients with advanced biliary tract carcinoma: results of the 40955 EORTC trial. Eur J Cancer 41: 398–403 15691639 10.1016/j.ejca.2004.10.026

[bib4] Khan SA, Davidson BR, Goldin R, Pereira SP, Rosenberg WM, Taylor-Robinson SD, Thillainayagam AV, Thomas HC, Thursz MR, Wasan H (2002) Guidelines for the diagnosis and treatment of cholangiocarcinoma: consensus document. Gut 51(Suppl 6): VI1–VI9 12376491 10.1136/gut.51.suppl_6.vi1 PMC1867742

[bib5] Khan SA, Thomas HC, Davidson BR, Taylor-Robinson SD (2005) Cholangiocarcinoma. Lancet 366: 1303–1314 16214602 10.1016/S0140-6736(05)67530-7

[bib6] Kornek GV, Schuell B, Laengle F, Gruenberger T, Penz M, Karall K, Depisch D, Lang F, Scheithauer W (2004) Mitomycin C in combination with capecitabine or biweekly high-dose gemcitabine in patients with advanced biliary tract cancer: a randomised phase II trial. Ann Oncol 15: 478–483 14998852 10.1093/annonc/mdh096

[bib7] Lazcano-Ponce EC, Miquel JF, Munoz N, Herrero R, Ferrecio C, Wistuba II, Alonso de Ruiz P, Aristi Urista G, Nervi F (2001) Epidemiology and molecular pathology of gallbladder cancer. CA Cancer J Clin 51: 349–364 11760569 10.3322/canjclin.51.6.349

[bib8] Louvet C, de Gramont A, Tournigand C, Artru P, Maindrault-Goebel F, Krulik M (2001) Correlation between progression free survival and response rate in patients with metastatic colorectal carcinoma. Cancer 91: 2033–2038 11391582

[bib9] Misra MC, Guleria S (2006) Management of cancer gallbladder found as a surprise on a resected gallbladder specimen. J Surg Oncol 93: 690–698 16724357 10.1002/jso.20537

[bib10] Moufarij MA, Phillips DR, Cullinane C (2003) Gemcitabine potentiates cisplatin cytotoxicity and inhibits repair of cisplatin-DNA damage in ovarian cancer cell lines. Mol Pharmacol 63: 862–869 12644587 10.1124/mol.63.4.862

[bib11] Pandey M, Krishnan Nair C (2004) Chemotherapy for gallbladder cancer (Protocol). The Cochrane Database of Systematic Reviews Issue 1, Art No.: CD004546. DOI: 10.1002/14651858.CD004546

[bib12] Patel T (2006) Cholangiocarcinoma. Nat Clin Pract Gastroenterol Hepatol 3: 33–42 16397610 10.1038/ncpgasthep0389

[bib13] Rajagopalan V, Daines WP, Grossbard ML, Kozuch P (2004) Gallbladder and biliary tract carcinoma: a comprehensive update, Part 1. Oncology (Huntingt) 18: 889–896 15255172

[bib14] Randi G, Franceschi S, La Vecchia C (2006) Gallbladder cancer worldwide: geographical distribution and risk factors. Int J Cancer 118: 1591–1602 16397865 10.1002/ijc.21683

[bib15] Rao S, Cunningham D, Hawkins RE, Hill ME, Smith D, Daniel F, Ross PJ, Oates J, Norman AR (2005) Phase III study of 5FU, etoposide and leucovorin (FELV) compared to epirubicin, cisplatin and 5FU (ECF) in previously untreated patients with advanced biliary cancer. Br J Cancer 92: 1650–1654 15856037 10.1038/sj.bjc.6602576 PMC2362051

[bib16] van Moorsel CJ, Pinedo HM, Veerman G, Bergman AM, Kuiper CM, Vermorken JB, van der Vijgh WJ, Peters GJ (1999) Mechanisms of synergism between cisplatin and gemcitabine in ovarian and non-small-cell lung cancer cell lines. Br J Cancer 80: 981–990 10362105 10.1038/sj.bjc.6690452 PMC2363050

